# Supplementary material for: Reparative Efficacy of Liposome-Encapsulated Oleanolic Acid against Liver Inflammation Induced by Fine Ambient Particulate Matter and Alcohol in Mice
Source: Pharmaceutics. 2022 May 23;14(5):1108. doi: 10.3390/pharmaceutics14051108 (PMC9148131; doi:10.3390/pharmaceutics14051108)
Supplement: Supplementary file 1 [file pharmaceutics-14-01108-s001.zip › pharmaceutics-1701380-supplementary.pdf]

Supplement figures

a

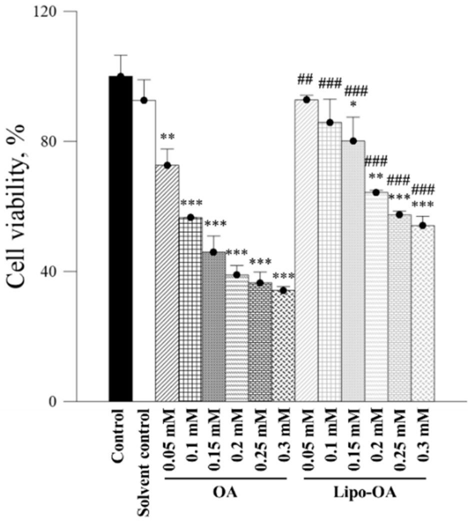

b

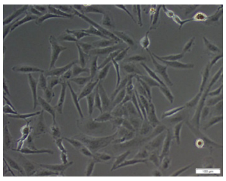
Control

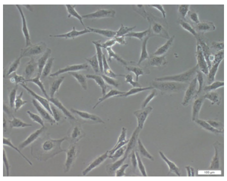
OA 0.05 mM

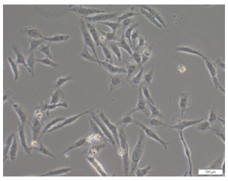
OA 0.1 mM

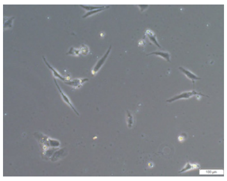
OA 0.15 mM

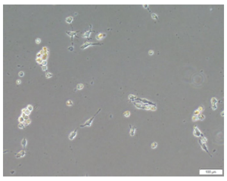
OA 0.2 mM

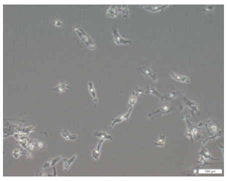
OA 0.25 mM

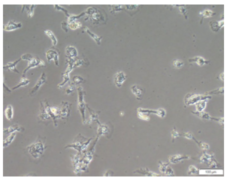
OA 0.3 mM

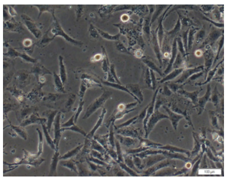
Lipo-OA 0.05 mM

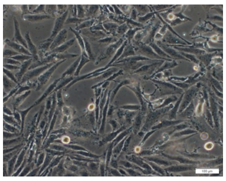
Lipo-OA 0.1 mM

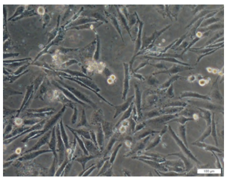
Lipo-OA 0.15 mM

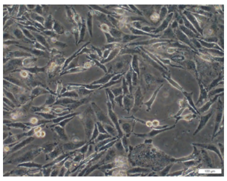
Lipo-OA 0.2 mM

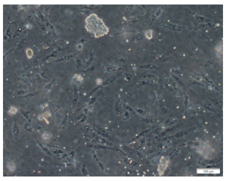
Lipo-OA 0.25 mM

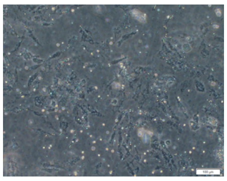
Lipo-OA 0.3 mM

c

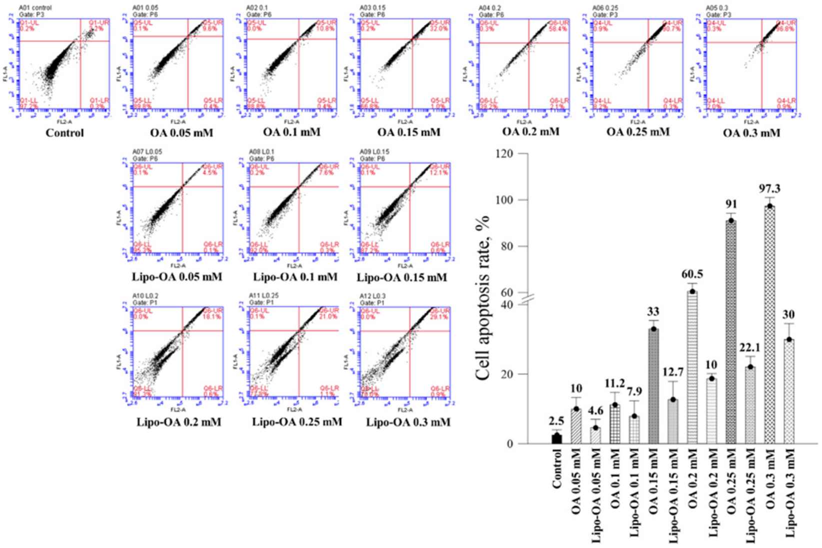

Figure S1. (**a**) MTT assay for OA and Lipo-OAs cytotoxicity. (**b**) Effects on LX-2 cell morphology monitored through light microscopy (magnification, 100×). (**c**) Flow cytometry assay for apoptosis in LX-2 cells treated with OA and Lipo-OAs at various concentrations. * p<0.05, ** p<0.01, *** p<0.001 compared with control group. ## p<0.01, ### p<0.001 compared with OA. Solvent control: DMSO solution that used to dissolve OA.

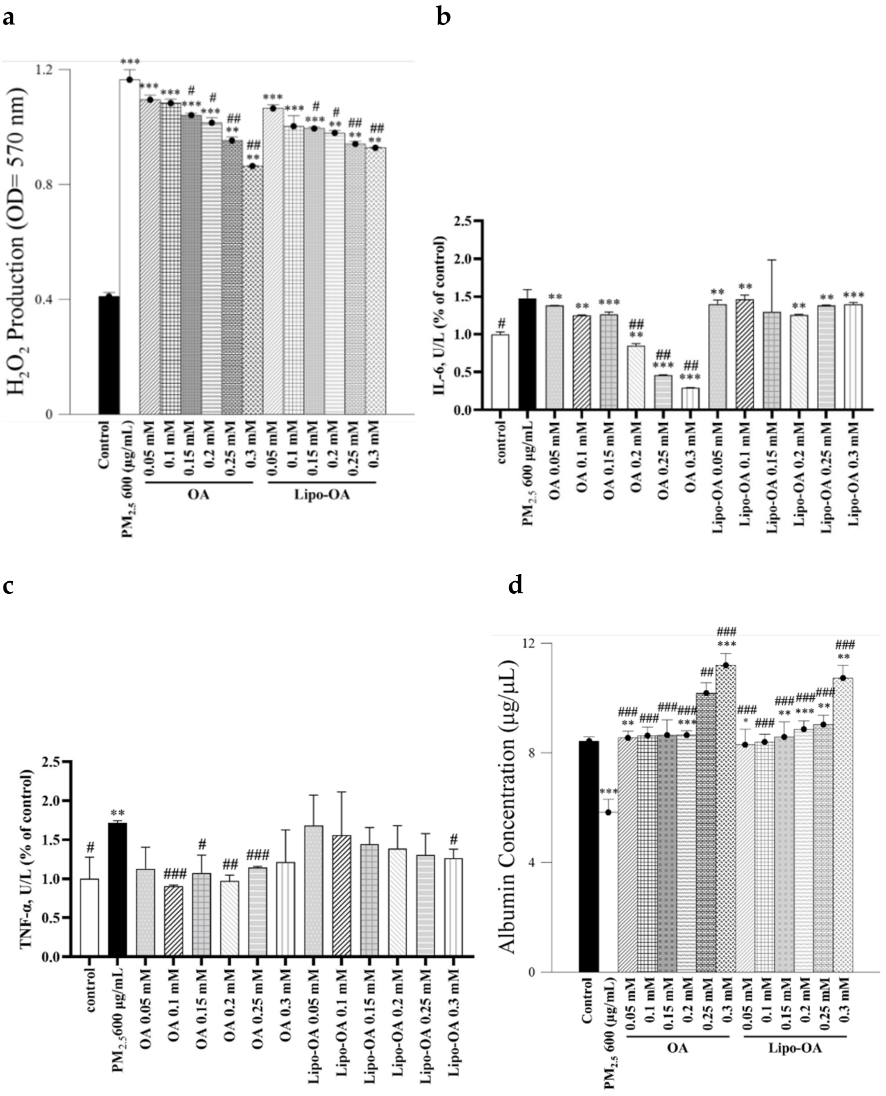

Figure S2. (**a**) Assessment of H_2O_2 scavenging activity. (**b**) IL-6 and (**c**) TNF-α response of LX-2 cells treated with $PM_{2.5}$ at various concentrations. (**d**) Albumin secretion by LX-2 cells with $PM_{2.5}$-induced inflammation after treatment with OA and Lipo-OAs at various concentrations. *p<0.05, **p<0.01, p***<0.001 compared with control group; # p<0.05, ##p<0.01, ###p<0.001 compared with $PM_{2.5}$ 600 µg/mL group.

a

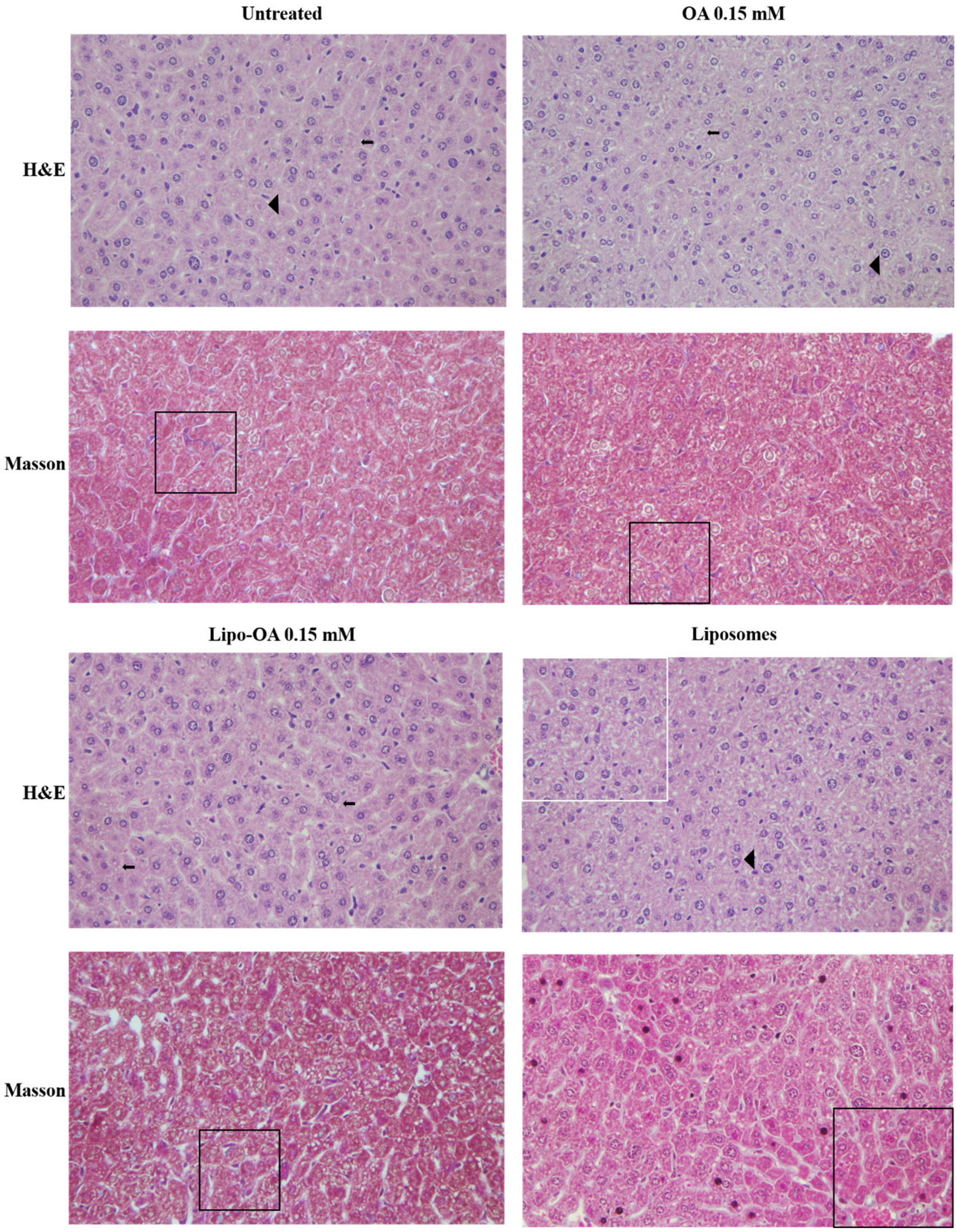

Untreated
OA 0.15 mM
H&E
Masson
Lipo-OA 0.15 mM
Liposomes
H&E
Masson

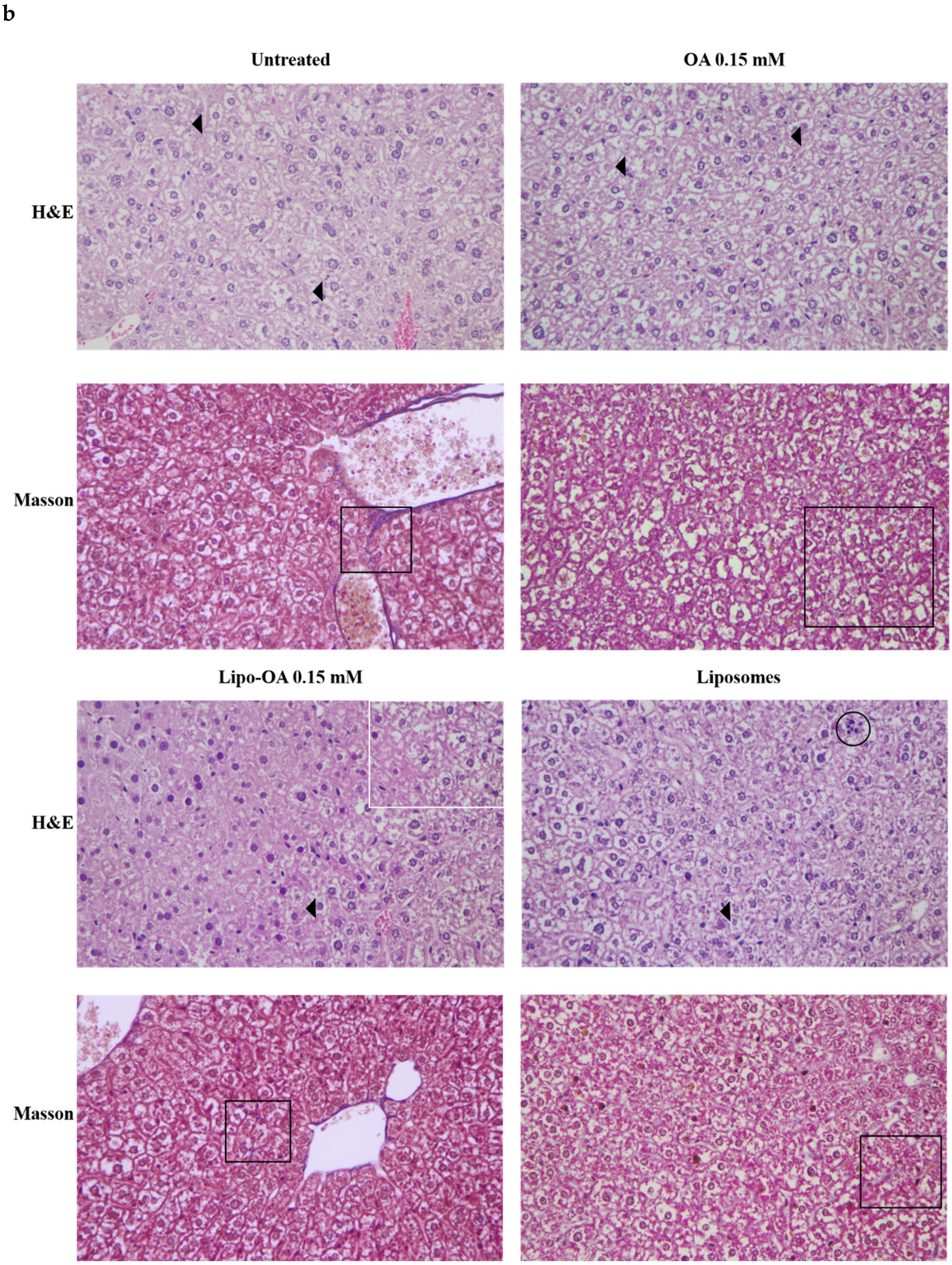

Figure S3. Hematoxylin and eosin (H&E) staining and Masson's trichrome staining of the sections of $PM_{2.5}$-induced inflammation liver treated with OA, Lipo-OAs, or pure liposomes (magnification, 200×): (a) after 4 treatment weeks, and (b) after 6 treatment weeks. The geometric figure was the representative area of: Patchy steatosis (white box), steatosis (black arrow), Mallory bodies and ballooning cells (black arrowhead), necrosis (black circle), single-cell apoptosis (black triangle), and pericellular fibrosis (black box).

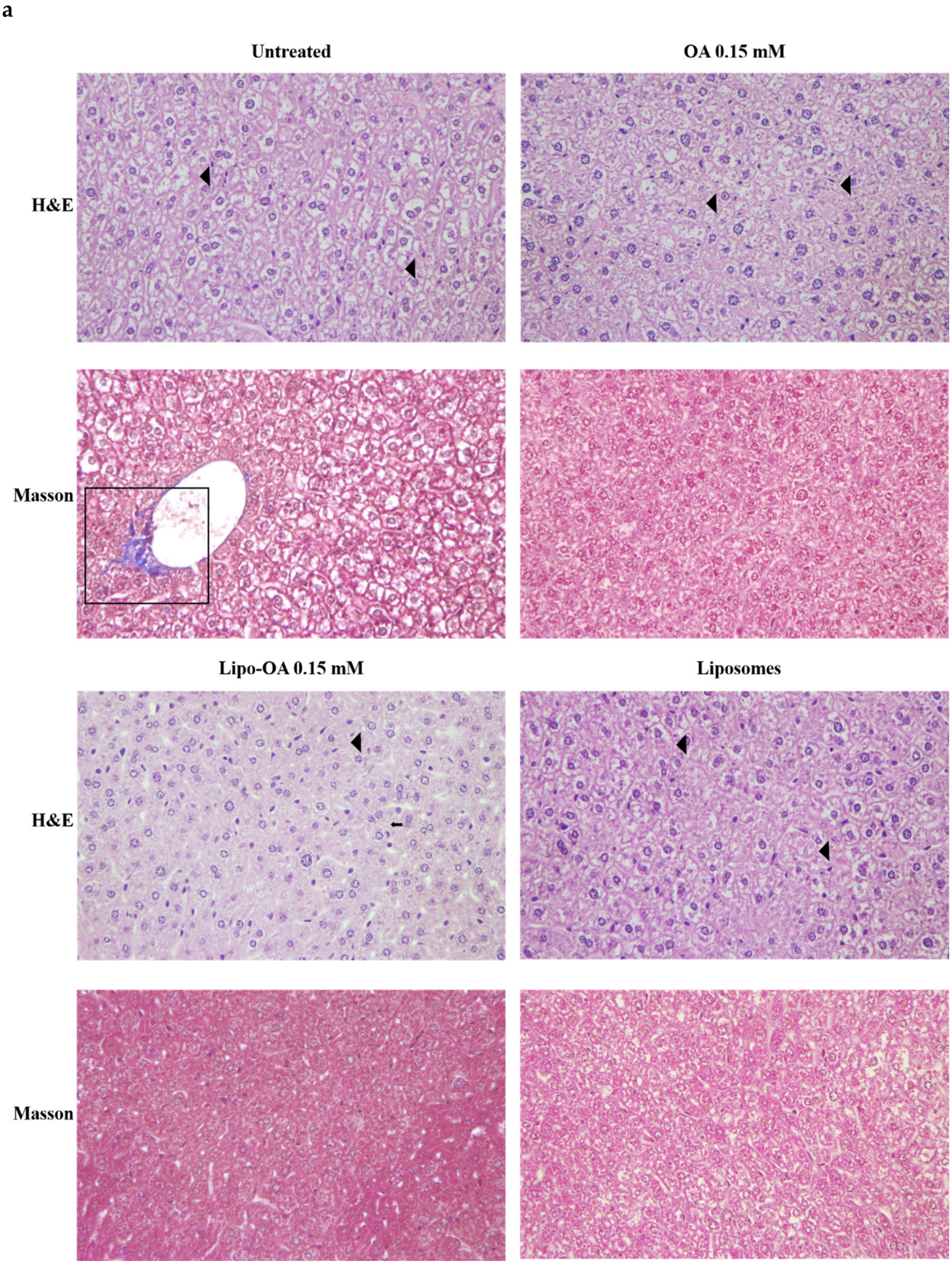
a
Untreated
OA 0.15 mM
H&E
Masson
Lipo-OA 0.15 mM
Liposomes
H&E
Masson

b

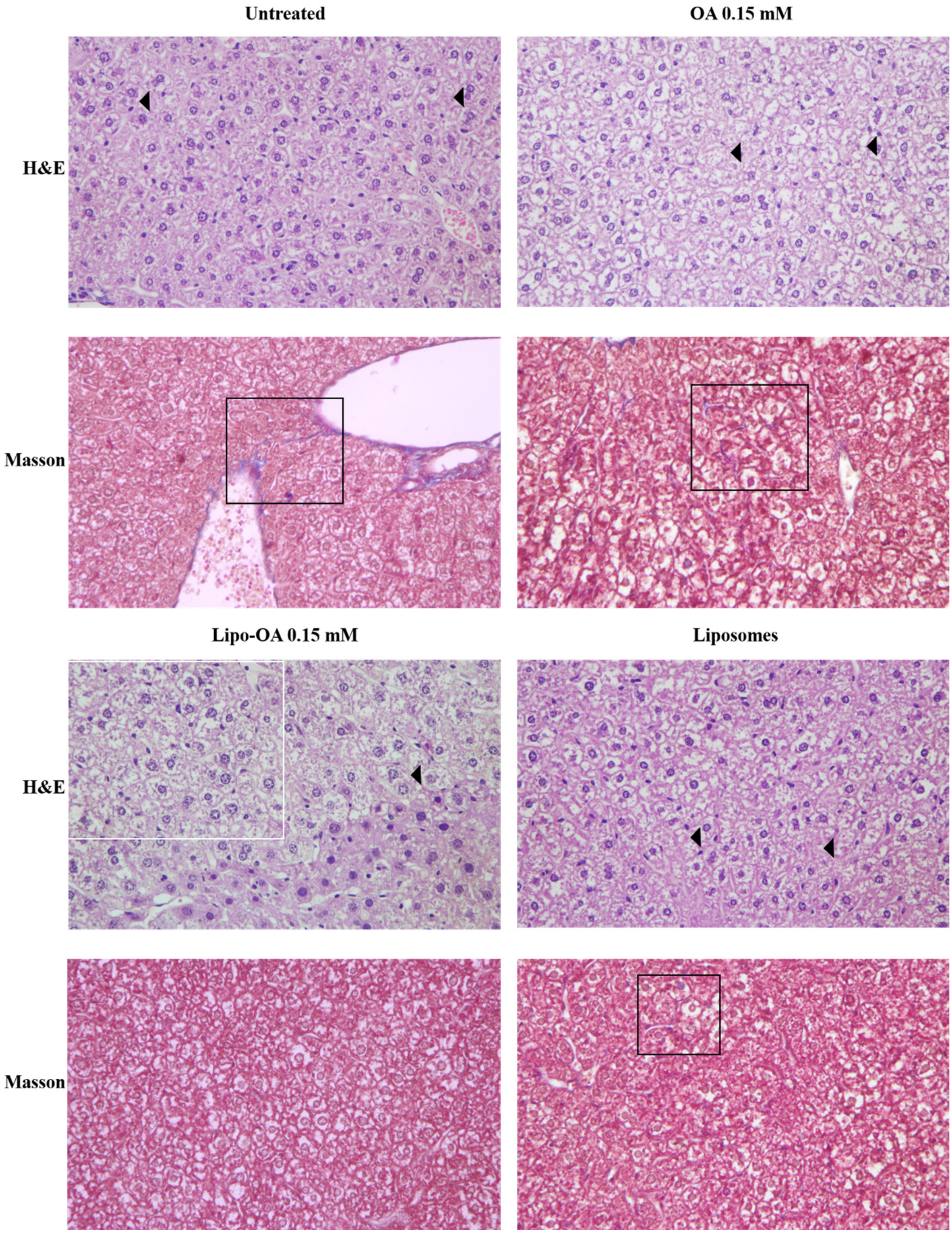

Figure S4. H&E staining and Masson's trichrome staining of the sections of $PM_{2.5}$ and alcohol–induced inflammation liver treated with OA, Lipo-OAs, or pure liposomes (magnification, 200×): after (**a**) 4 and (**b**) 6 treatment weeks. The geometric figure was the representative area of: Patchy steatosis (white box), steatosis (black arrow), Mallory bodies and ballooning cells (black arrowhead), necrosis (black circle), single-cell apoptosis (black triangle), and pericellular fibrosis (black box).
